# TRPV2: A Cancer Biomarker and Potential Therapeutic Target

**DOI:** 10.1155/2020/8892312

**Published:** 2020-12-10

**Authors:** Kodappully S. Siveen, Parveen B. Nizamuddin, Shahab Uddin, Mohamed Al-Thani, Michael Paul Frenneaux, Ibrahim A. Janahi, Martin Steinhoff, Fouad Azizi

**Affiliations:** ^1^Translational Research Institute, Academic Health System, Hamad Medical Corporation, Doha, Qatar; ^2^Public Health Department, Ministry of Public Health, Doha, Qatar; ^3^Division of Pediatric Pulmonology Sidra Medicine, Doha, Qatar; ^4^Department of Dermatology and Venereology, Hamad Medical Corporation, Doha, Qatar; ^5^Weill Cornell Medicine, Doha, Qatar; ^6^Weill Cornell University, New York, NY, USA

## Abstract

The Transient Receptor Potential Vanilloid type-2 (TRPV2) channel exhibits oncogenicity in different types of cancers. TRPV2 is implicated in signaling pathways that mediate cell survival, proliferation, and metastasis. In leukemia and bladder cancer, the oncogenic activity of TRPV2 was linked to alteration of its expression profile. In multiple myeloma patients, TRPV2 overexpression correlated with bone tissue damage and poor prognosis. In prostate cancer, TRPV2 overexpression was associated with the castration-resistant phenotype and metastasis. Loss or inactivation of TRPV2 promoted glioblastoma cell proliferation and increased resistance to CD95-induced apoptotic cell death. TRPV2 overexpression was associated with high relapse-free survival in triple-negative breast cancer, whereas the opposite was found in patients with esophageal squamous cell carcinoma or gastric cancer. Another link was found between TRPV2 expression and either drug-induced cytotoxicity or stemness of liver cancer. Overall, these findings validate TRPV2 as a prime candidate for cancer biomarker and future therapeutic target.

## 1. Introduction

Cancer patients could get a significant clinical benefit from the identification of molecular targets that play a polar role in tumor cell growth and survival, amenable to an approach with preciseness in patient medication. Nowadays, the discovery of new cancer therapies or improvement of current ones requires an understanding of the mechanism(s) of cancer progression and identification of biomarkers that are causally connected to instead of merely related to the disease process. The concept of precision medicine, which consists of identifying the molecular signature of individual tumors that can be selected for the most appropriate therapeutic approach, has become the pivot of contemporary oncology. On this basis, for biomarkers to assume their rightful role, they need to be befittingly altered by effective therapeutic interventions and modify the definition of the populations of patients who presumably will benefit from precision medicine.

The TRPV2 channel has attracted the attention in many deadly cancers as one of several candidate channels that are involved in the proliferation and resistance of tumor cells to apoptotic cell death. Depending on the type of cancer, different alterations in the TRPV2 gene (i.e., loss, gain, and splicing) were found to exhibit oncogenic capacity linked to a tumor's growth and metastasis. This review focuses on the pathophysiological significance of the TRPV2 channel in many kinds of cancers, and we hope to offer the reader a flavor of how the measurable molecular changes in TRPV2 could validate its quality as a cancer biomarker and potential therapeutic target.

## 2. TRPV2 Identity and Structure

*Other names:* the other names are VRL, VRL-1, and VRL1.

*HGNC (Hugo):* the approved symbol is TRPV2.

**Figure 1:**
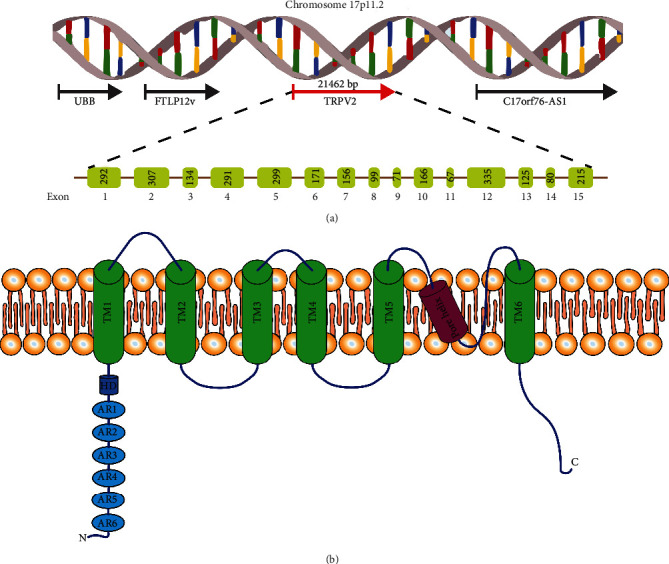
TRPV2 gene and protein structural organization. (a) Schematic representation of human TRPV2 and neighboring genes on the shorter arm of chromosome 17. UBB: ubiquitin B; FTLP12: ferritin light chain pseudogene 12; C17orf76-AS1: C17orf76 antisense RNA 1. (b) The monomeric structure of TRPV2 contains 761 amino acids, six transmembrane spanning domains (TM), and a pore helix region located between TM5 and TM6. Both N- and C-termini are cytosolic with the N-terminal having a hydrophobic domain (HD) and 6 ankyrin repeats (AR).

*Chromosome location:* the gene encoding TRPV2 is located on chromosome 17 (17p11.2), ~10 Mb apart from TRPV1, and colocalizes upstream with UBB (ubiquitin B) and FTLP12 (ferritin, light polypeptide pseudogene 12) and downstream with C17orf76-AS1 (C17orf76 antisense RNA 1) (nonprotein coding) (see Figure 1(a)).

*DNA/RNA:* the TRPV2 gene consists of 15 exons and 14 introns spanning 21462 bp of DNA and includes 5′-/3′-noncoding regions. The TRPV2 mRNA product length is 2829 bp, and analysis of its alternative splice variants showed the existence of a larger 2.3 kbp product as the full-length TRPV2 (f-TRPV2) and a smaller 1.9 kbp product as a novel splice variant of TRPV2 (s-TRPV2) lacking exons 10 and 11, corresponding to the pore region and the fifth and sixth transmembrane domains [1].

*Protein:* TRPV2 functions as a tetramer, with each monomer containing 761 residues, with a large N-terminal region of 389 amino acids, a smaller 250-residue transmembrane (TM) domain, and a 122-residue C-terminal region. The channel structure, recently resolved by cryoelectron microscopy [2, 3], shows six transmembrane spanning domains, a putative highly conserved pore-loop region located between transmembrane domains 5 and 6, and cytoplasmic amino and carboxy termini [4, 5]. The transmembrane segment 6 (S6) is involved in gate opening with a rotation of the ankyrin-repeat domain coupled with pore opening via the TRP domain [3, 5]. The 3D structure of TRPV2 reveals “hanging gondola architecture” with a calmodulin-binding site present in the C-terminal of TRPV2 (654-683) and six ankyrin repeats present in the N-terminal tail and may play a role in the interaction between subunits of TRPV2 [6] (see Figure 1(b)).

## 3. TRPV2 Regulation

**Figure 2:**
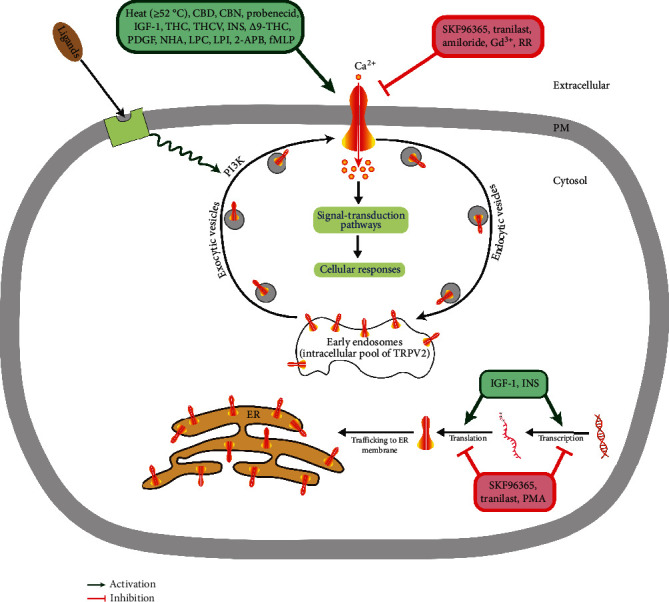
TRPV2 regulation is involved in cellular responses relevant to cancer. In unstimulated cells, the majority of TRPV2 resides in the endoplasmic reticulum and (early) endosomal compartments, while a minor fraction of the channel is present at the cell surface. Upon stimulation of the cells with a phosphatidylinositol 3-kinase- (PI3K-) activating ligand, the vesicular transport of TRPV2 is increased between intracellular compartments and plasma membrane. Subsequently, the increase in TRPV2-mediated Ca^2+^ entry leads to activation of signaling pathways involved in cellular processes (e.g., survival, apoptosis, proliferation, differentiation, and migration/metastasis). When stimulation is terminated, TRPV2 is recycled back to the intracellular compartments via an endocytic pathway. PM: plasma membrane; ER: endoplasmic reticulum.

TRPV2 is a homotetrameric N-glycosylated protein that is largely located in the endoplasmic reticulum compartment under unstimulated conditions. However, a ligand (mechanical stress) that stimulates the activity of phosphatidylinositol 3-kinase (PI3K) triggers TRPV2 translocation to the plasma membrane, where it functions as a cation channel (Figure 2). The putative (exogenous) activators and inhibitors of TRPV2 are listed in Table 1. Patch-clamp electrophysiology showed a nonselective permeability (*P*) of TRPV2 to Ca^2+^>Mg^2+^>Na^+^~Cs^+^~K^+^ (*P*_Ca^2+^_/*P*_Na^+^_ = 2.94; *P*_Mg^2+^_/*P*_Na^+^_ = 2.40) [4]. The ratio of the functional full-length TRPV2 isoform (f-TRPV2) and the short splice variant (s-TRPV2) that is poreless and nonfunctional appears to control the biological function and oncogenicity of TRPV2 [5, 7].

## 4. TRPV2 Expression Profile Is Altered in Hematological Cancers

In immune cells, TRPV2 acts as a molecular sensor in diverse functions that include phagocytosis/degranulation [5, 8], migration/chemotaxis [5, 8–10], cytokine secretion [8], infiltration of tissues [11], cytokine release [12], endocytosis [13], inflammasome activity [14], neuroinflammation [15], and podosome assembly [16]. Indeed, circulating lymphocytes are subjected to fluid flow, changes in osmolarity and blood pressure, changes of shape during processes such as extravasation/infiltration of tissues, antigen recognition, and maturation/activation. TRPV2 may facilitate these processes as it is directly or indirectly gated by mechanical stretch [17].

Loss, gain, or mutation of the TRPV2 gene have been reported in hematological tumors, including mantle cell lymphoma, multiple myeloma, Burkitt lymphoma, acute myeloid leukemia, and myelodysplastic syndrome [18, 19]. Furthermore, TRPV2 expression in CD34^+^/CD45^+^/CD133^+^/CD73^+^ hematopoietic stem cells, from which all lineages of blood cells are derived, suggests a role for this channel in hematopoietic cell-derived tumors, i.e., leukemias and lymphomas [20].

### 4.1. TRPV2 Oncogenicity in Leukemia Is Driven by Alteration of Its Transcription Profile

A strong piece of evidence for the involvement of TRPV2 in the pathogenesis of leukemia and associated pulmonary dysfunction comes from our recent study revealing that TRPV2 mRNA transcripts and protein expression profiles are altered in leukemic blasts (LBs) [21]. Indeed, the full-length glycosylated TRPV2 protein (f-TRPV2) and a short splice variant of TRPV2 (s-TRPV2) had opposite trends of expression in LBs compared to normal peripheral blood mononuclear cells (PBMCs). In LBs, the oncogenic isoform f-TRPV2 was more abundant than the nononcogenic variant s-TRPV2, but the opposite was observed in PBMCs. The oncogenic activity of TRPV2 was demonstrated by silencing its expression, resulting in cell cycle arrest and apoptotic cell death. SKF96365 and tranilast, known to target TRPV2 Ca^2+^ activity, altered the expression profiles of this channel's isoforms and exhibited anticancer properties in LBs. Furthermore, this study showed that targeting TRPV2 could affect the signaling pathways associated with chemotaxis/infiltration processes, prompting the assessment of TRPV2 as a potential pharmacodynamic biomarker especially in the setting where leukemia might be associated with a high risk of organ (lung) infiltration by LBs.

### 4.2. TRPV2 Overexpression Correlates with Bone Lesions and Poor Prognosis in Multiple Myeloma Patients

Multiple myeloma (MM) is caused by the accumulation of a malignant plasma cell (PC) monoclonal population in the bone marrow (BM). Immunohistochemistry revealed overexpression of TRPV2 in BM biopsies collected from patients with MM compared to normal BM [22]. An additional analysis of publically available gene expression data of BM plasma cells from MM counterparts evidenced significantly a higher transcriptional level of TRPV2 in plasma cells of patients with shorter Event-Free Survival (EFS, <24 months) compared to patients with longer EFS (≥24 months) [22]. Notably, TRPV2 overexpression correlated with a poor prognosis in MM patients due to enhancement of the interaction between myeloma cells and bone marrow stromal cells by the channel's Ca^2+^ activity, causing osteoclast-mediated bone destruction. In fact, in MM cells, switching from low to high Ca^2+^ conditions activated TRPV2 and induced osteoclastogenesis via the Ca^2+^-calcineurin-NFATc3 signaling pathway, ultimately leading to excessive secretion of inflammatory cytokines and nuclear factor kappa *β* (NF-*κβ*) ligand (RANKL), which contributed to the progression of osteoclastic differentiation (formation of osteoclasts). Interestingly, this cascade of events was suppressed when TRPV2 Ca^2+^ influx activity was blocked by SKF96365 [22]. In another study, TRPV2 stimulation by CBD decreased MM cell proliferation and increased sensitivity to drug-induced cell death [23]. Phenotypic analysis of MM patient samples identified the presence of two PC populations CD138^+^TRPV2^+^ and CD138^+^TRPV2^−^, whereas only the CD138^+^TRPV2^−^ population was present in RPMI8226 and U266 MM cell lines. MM cells with a CD138^+^TRPV2^+^ phenotype were more sensitive to CBD alone or a combination of CBD and bortezomib than MM cells with a CD138^+^TRPV2^−^ phenotype. Inhibition of growth, cell cycle arrest, and triggering of MM cell death involved regulation of the ERK, AKT, and NF-*κβ* pathways with major effects in TRPV2-overexpressing cells [23].

## 5. Detrimental or Beneficial: The Role of TRPV2 in Brain Cancer

All the evidence points to negative control of the survival and proliferation of glioblastoma (GBM) by TRPV2 [24–26]. In fact, a progressive decline in TRPV2 expression was observed as the histological stage of the disease increased [24, 26]. TRPV2 silencing increased proliferation and resistance to apoptosis in the high-grade U87MG glioma cell line, which displays a predominant mesenchymal stem cell (MSC) phenotype [24], whereas overexpression of TRPV2 enhanced differentiation of glioblastoma stem-like cells (GSCs) and reduced tumor size and viability [27]. Indeed, the control of GSC phenotype by TRPV2-mediated Ca^2+^ activity was shown to determine the fate of GBM. TRPV2 silencing or inhibition with RR markedly reduced the expression of differentiation markers glial fibrillary acidic protein (GFAP) and class III *β*-tubulin in GSCs. Similarly, PMA treatment reduced TRPV2 expression levels, inhibited astroglial differentiation, and promoted GSC proliferation. On the other hand, overexpression of TRPV2 promoted the differentiation and inhibited the proliferation of GSCs. The injection of TRPV2-overexpressing GSCs in a xenograft mouse model reduced tumor growth due to cell cycle arrest and increased glial differentiation [27].

TRPV2 activation was reported to promote GSC differentiation and inhibition of gliomagenesis [27–29]. For example, the TRPV2 agonist CBD was shown to induce GSC differentiation by inhibiting their clonogenic capacity [27, 30, 31]. In fact, treatment of GSCs with CBD triggered downregulation of genes involved in chemoresistance (e.g., BCL-XL and CTDS) and upregulation of genes involved in apoptosis (e.g., BAD and BAX) [32]. Moreover, CBD induced glioma cell chemosensitivity and apoptosis by increasing uptake of the cytotoxic drug doxorubicin [27, 33]. CBD was also found to increase GSC chemosensitivity to other conventional anticancer drugs such as carmustine (BCNU) [26, 34]. Combining Temozolomide (TMZ) with THC or with THC plus CBD was reported to substantially reduce tumor growth in glioma xenografts [35]. The pore region of TRPV2, critical for its Ca^2+^ activity, was required for boosting glioma chemosensitivity to cytotoxic drugs [34]. In patients with high-grade glioma, CBD has been administered in conjunction with chemoradiation therapy to improve outcome and survival [36]. In this regard, CBD was found to upregulate the expression of the transcription factor acute myeloid leukemia-1 (AML-1A) isoform, which is involved in GSC differentiation. Interestingly, the downregulation of AML-1A, which binds TRPV2 gene promoters, led to the restoration of the undifferentiated (immature) phenotype of mature GSCs [26, 37]. In particular, the establishment of the TRPV2 interactome [38] raised questions and issues and also opened opportunities to discover new biomarkers and therapeutic targets in GBM. The strongest interactors with TRPV2 were ABR, ARL15, NTM, Opalin, SACM1L, and ST18 proteins [38]. A high TRPV2 interactome protein expression correlated with greater tumor progression, recurrence and TMZ resistance, and poor prognosis of GBM patients [38]. Therefore, the TRPV2 interactome-based signature permits discrimination between high- and low-risk GBM, in terms of overall survival, with less survival in GBM patients expressing the TRPV2 protein interactome.

## 6. TRPV2 Activation Is a Potential Therapeutic Strategy for Breast Cancer

In early *in vitro* studies, tranilast was shown to inhibit the proliferation of several human breast cancer cell lines (e.g., MCF-7, BT-474, and MDA-MB-231) [39–41] and rat mammary carcinoma stem cell LA7 [41]. In a xenograft model of mouse mammary carcinoma cell line 4T1, tranilast significantly reduced the growth of the primary tumor and effectively blocked its metastasis into the lungs and liver [41]. In particular, tranilast was reported to block IGF-1-induced cell growth of the low TRPV2-expressing breast cancer cell line MCF-7 by blocking calcium influx mediated by a voltage-independent calcium-permeable channel [40]. However, TRPV2 activation by the antimicrobial peptide LL-37 promoted migration (metastasis) of the highly TRPV2-expressing breast cancer cell line MDA-MB-435 and low TRPV2-expressing MCF-7 and MDA-MB-231 cells [42]. In MDA-MB-435 cells, LL-37 acted by raising intracellular calcium through PI3K/AKT-dependent recruitment and activation of the TRPV2 channel. TRPV2 silencing abrogated LL-37-induced migration of all cell lines tested [42]. Curiously, this peptide is a molecule with multiple physiological functions and derived from the C-terminus of the human cationic antimicrobial protein (hCAP18) found abundantly expressed in epithelial cells. A puzzling finding in this study is that a large panel of human breast tumors was tested positive for LL-37 expression and correlated with TRPV2 protein expression. LL-37 was found to stimulate both cell proliferation and migration in multiple forms of cancer [43] and perhaps may involve TRPV2 activation. When TRPV2 channel stimulation is coupled with chemotherapy, the outcome is a reduction in breast tumor growth as recently demonstrated [44]. A higher recurrence-free survival was associated with greater expression of TRPV2 in triple-negative breast cancer (TNBC) and estrogen receptor *β*- (ER*β*-) negative breast cancer patients who underwent a chemotherapy regimen with doxorubicin [44]. In fact, TRPV2 activation with CBD enhanced the sensitivity of breast cancer cells towards the chemotherapeutic drug, resulting in greater inhibition of tumor growth *in vitro* and *in vivo* [44]. Therefore, the therapeutic approach taken for modulating TRPV2 expression should be carefully evaluated to obtain a beneficial outcome for breast cancer patients.

## 7. TRPV2 Overexpression Correlates with Low Survival in Patients with Esophageal Cancer

Patients with esophageal squamous cell carcinoma (ESCC) harboring high expression levels of TRPV2 had a worse five-year overall survival rate after surgery when compared to patients with low TRPV2 expression [45]. The highest level of TRPV2 expression was detected in advanced stages of the disease and metastatic ESCC in lymph nodes. TRPV2 was overexpressed at both mRNA and protein levels in ESCC cell lines. In particular, knockdown of TRPV2 decreased cell proliferation, cell cycle progression, and migration (invasion). Moreover, microarray analysis demonstrated that TRPV2 silencing caused downregulation of WNT/*β*-catenin signaling-related genes and basal cell carcinoma signaling-related genes [46]. Notably, tranilast was found to target the TRPV2-overexpressing dehydrogenase 1-positive cancer stem cells (CSCs) isolated from the TE8 ESCC cell line. Therefore, the downregulation of TRPV2 could be a good therapeutic strategy to eradicate the stemness potential of ESCC [47].

## 8. TRPV2 Overexpression Is a Proliferation Marker in Gastric Cancer

Gastric cancer was classified as the third most common deadly cancer in 2018, just after lung and colorectal cancer [48]. During early-stage gastric cancer, most patients are asymptomatic, and a diagnosis is often made when the cancer is at an advanced stage and shows metastasis [49]. The potential implication of TRPV2 in the pathology of gastric cancer (GC) was inferred from a study analyzing the association between the expression of intracellular calcium regulator genes (CaRGs) and different clinicopathological parameters in a large collection of GC patients using an integrated bioinformatic data-processing procedure [50]. A high expression level of TRPV2 was significantly associated with a shorter overall survival suggesting that TRPV2 could be indicative of the advanced stage of GC. The most impressive finding was that TRPV2 protein was not detected in normal stomach mucosa tissue sections, whereas its expression in tumor samples ranged mostly from medium to high levels. TRPV2 expression was also linked to a poor outcome in Lauren's intestinal-type GC and patients under adjuvant care. This study highlighted the clinical relevance of TRPV2 as a prognostic biomarker and therapeutic target to improve the management of GC.

## 9. TRPV2 Channel Is Linked to Stemness Features in Liver Cancer

TRPV2 involvement in human hepatocarcinogenesis was inferred from the observation that TRPV2 expression at mRNA and protein levels is increased in moderately and well-differentiated hepatocellular carcinoma tissues compared to poorly differentiated tumors. Moreover, a correlation between TRPV2 expression and portal vein invasion was confirmed [51]. Additional studies have demonstrated a link between TRPV2 expression and either drug-induced cytotoxicity [52] or the stemness of liver cancer. In the human hepatocellular carcinoma cell lines HepG2 and Huh-7, TRPV2 was found to mediate H_2_O_2_-induced oxidative stress and cell death by potentiating the inhibition of the prosurvival signaling proteins (Akt, Nrf2) and enhancing prodeath signaling proteins (p38, JNK1) [52]. The hepatocellular carcinoma phenotype is driven by liver cancer stem-like cell (LCSLC) subpopulations CD133^+^, CD90^+^, CD44^+^, CD13^+^, and CD24^+^, which are endowed with self-renewal [53]. Interestingly, in the liver cancer cell line HepG2, shRNA-based TRPV2 knockdown promoted the expression of stem cell markers (i.e., CD133, CD44, and ALDH1), spheroid, and colony formation. Opposite effects were observed in SMMC-7721 cells overexpressing exogenous TRPV2. Similarly, the TRPV2 antagonist tranilast induced expression of liver cancer stem-like cell (LCSLC) markers and led to spheroid and colony formation, whereas the TRPV2 agonist probenecid produced an opposite effect on liver cancer cell lines [54]. In line with these findings, probenecid inhibited while tranilast promoted tumor growth of HepG2 xenografts in the severe combined immunodeficiency (SCID) mouse model. Notably, reduction of TRPV2 mRNA and protein expression levels in poorly differentiated tumors in comparison to higher differentiated hepatoma [53] supports the idea that reduced TRPV2 expression promotes the stem cell features of hepatoma cells during the early stages of tumor development [55]. Therefore, there is a real therapeutic potential based on the positive-loop regulation of TRPV2 to eradicate the highly tumorigenic LCSLCs [55].

## 10. Alteration of TRPV2 Expression Profile Aggravates Bladder Cancer

The oncogenic activity of the TRPV2 channel was first discovered in bladder carcinoma [1] and was attributed to alteration of the channel transcription profile in urothelial carcinoma tissues and cell lines. In fact, f-TRPV2 and s-TRPV2 had opposite trends of expression in urothelial carcinoma tissues when compared to normal bladder specimens. During the progression of bladder cancer from low (pTa, pT1, and pT2) to higher more aggressive (pT3 and pT4) grades, f-TRPV2 and s-TRPV2 expression levels increased and decreased, respectively [1]. Nevertheless, the mechanism by which TRPV2 contributes to the pathogenesis of bladder cancer (BC) is not well understood. CBD triggered Ca^2+^ influx and apoptosis in the cell line T24, which represents a model of a poorly differentiated and high-grade human bladder carcinoma [56]. In contrast, such effect was not observed on the low-grade and highly differentiated human bladder carcinoma cell line RT4 treated with this TRPV2 agonist [56]. Similarly, treatment of the mouse bladder carcinoma cell line MBT-2 with TRPV2 agonists 2-APB or LPC induced Ca^2+^ influx and inhibited cell proliferation, but TRPV2 silencing induced an opposite effect [57]. TRPV2 activation was also shown to promote bladder cancer cell metastasis via either matrix metalloproteinase-2- (MMP-2-) [58] or adrenomedullin-dependent [59] mechanisms in the human bladder carcinoma cell line T24/83.

The abundance of the s-TRPV2 variant in normal and low-grade bladder cancer tissues suggests that it may function as a regulator of f-TRPV2 isoform [18]. Interestingly, s-TRPV2 was found to inhibit f-TRPV2-dependent cell migration by blocking recruitment of f-TRPV2 to the plasma membrane and its Ca^2+^ permeability [1, 9, 60]. This regulation is lost as cancer progresses into more aggressive and invasive stages, where clear evidence points to a key role of f-TRPV2 in the metastatic process [9, 57–59] perhaps by responding to excessive mechanical stress resulting from cell migration. In fact, f-TRPV2 properties as an endogenous sensor of noxious heat and mechanical stretch [1, 4, 5] are in line with a sensory role in urothelium [1]. The highly expressed IGF-1/IGF-1 receptor (IGF-1R) in bladder tumors [61] may team up with TRPV2 to respond to the high metabolic demands during the aggressive late stages of the disease. In conclusion, TRPV2 negatively regulates bladder cancer cell proliferation and could be a potential therapeutic target for the treatment of bladder cancer.

## 11. TRPV2 Overexpression Promotes Metastasis in Prostate Cancer

All current evidence points to a role of TRPV2 in the process of prostate cancer (PCa) metastasis. Overexpression of TRPV2 was not observed in stationary primary solid tumors at stages T2a and T2b, but it was noticeable in metastatic tumors at stage M1 as well as in metastatic PCa cell lines PC3 and DU-145 derived from the bone and the brain, respectively [62, 63]. The evaluation of TRPV2 as a key player in prostate cancer cell migration rather than cell growth was undertaken by *in vitro* as well *in vivo* studies. TRPV2 silencing with siRNA technology decreased PCa cell migration without affecting cell proliferation [62]. Similarly, TRPV2 silencing reduced metastasis of PC3-derived tumors established in nude mouse xenografts. In parallel, there was a decrease in the expression level of metastasis promoting proteases (i.e., cathepsin B, MMP-2, and MMP-9) [63]. In contrast, TRPV2 overexpression in PCa cells boosted cell migration and markers of invasion MMP-9 and cathepsin B [62]. Mechanistically, TRPV2 translocation/activity and actin reorganization fuel the migration process of PCa cells [64]. In fact, lysophospholipids (i.e. LPC and LPI) were reported to promote the migration of PC3 cells through the TRPV2/PI3K signaling pathway [62]. As in bladder cancer, adrenomedullin induced TRPV2-dependent PCa cell metastasis to the bone by affecting the receptor activator of NF-*κβ* ligand (RANKL) [59, 65]. Similarly, protein kinase-A-dependent calcitonin and calcitonin gene-related peptide were reported to promote bone metastasis in the same study. The deletion of the PDZ-binding domain of the calcitonin receptor abrogates the bone metastasis in an orthotopic PCa model [65]. In conclusion, these studies indicate TRPV2 to be a promising target to treat prostate cancer and metastasis.

## 12. TRPV2 Is a Potential Player in Skin Cancer

The role of TRPV2 in skin cancer remains uncertain since staining and semiquantitative analysis of skin samples taken from patients suffering from squamous cell carcinoma (SCC) or basal cell carcinoma (BCC) do not show a significant difference in protein in atypical keratinocytes [66]. Nevertheless, TRPV2 activation with 2-APB triggered necrosis and apoptosis in the human metastatic melanoma cell line A2058 [67].

## 13. Conclusions

**Figure 3:**
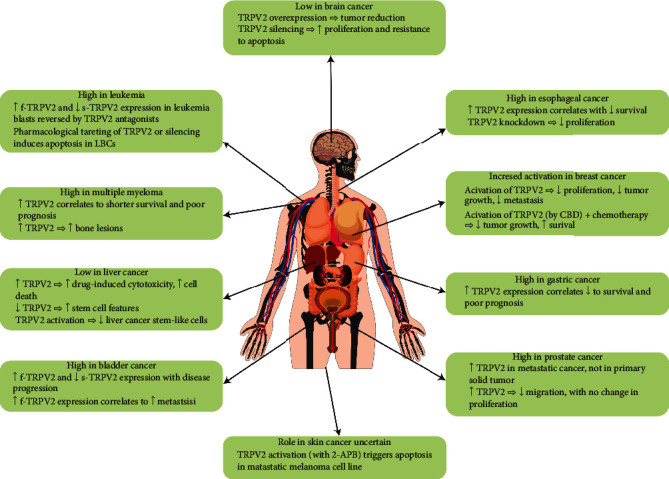
Schematic view summarizing organ tissues implicating TRPV2 deregulation in cancer.

The oncogenic activity of TRPV2 in cancer is mainly associated with the deregulation of its expression and/or alteration of its transcription profile. TRPV2 is involved in both early and late (advanced) stages of tumor development as well as metastasis. The eradication of some types of cancer (e.g., leukemia and bladder) requires induced loss or inhibition of TRPV2, whereas the suppression of other cancers (e.g., glioblastoma and breast) involves TRPV2 overexpression or activation (Figure 3). Though the possibility to use TRPV2 in cancer therapy is still in infancy, TRPV2 represents a novel promising pharmacologic/molecular target, especially in the management of the most aggressive metastatic cancers. For example, tranilast (INN, brand name Rizaben) already an approved drug for allergic diseases and with known and limited side effects could be a good candidate anticancer agent for patients with leukemia especially during the aggressive stages of the disease, which are associated with a high risk of infiltration of leukemic blasts into organs and inflammation. Clinical studies are needed to identify cancer populations that may benefit from TRPV2 as a molecular biomarker and perhaps will open new prospectives to explore TRPV2 as a novel therapeutic target in cancer and metastasis.

## Figures and Tables

**Table 1:** TRPV2 agonists and antagonists.

	Activators	Inhibitors
Activity	Heat (≥52°C), CBD, CBN, probenecid, IGF-1, THC, THCV, INS, *Δ*9-THC, PDGF, NHA, LPC, LPI, 2-APB, fMet-Leu-Phe (fMLP)	SKF96365, tranilast, amiloride, Gd^3+^, RR
Traffick^∗^	CBD, IGF-1, INS, LPC, LPI, PDGF, NHA, fMet-Leu-Phe (fMLP)	
Expression	IGF-1, INS	SKF96365, tranilast, PMA

^∗^Translocation of TRPV2 from cytoplasm to plasma membrane. CBD: cannabidiol; THC: (-)-trans-*Δ*9-tetrahydrocannabidol; CBN: cannabinol; THCV: *Δ*9-tetrahydro-cannabivarin; NHA: neuropeptide head activator; PDGF: platelet-derived growth factor; IGF-1: insulin-like growth factor-1; PMA: phorbol-12-myristate-13-acetate; RR: ruthenium red; Gd^3+^: gadolinium; LPC: lysophosphatidylcholine; LPI: lysophosphatidylinositol; 2-APB: 2-aminoethoxydiphenyl borate; fMLP: fMet-Leu-Phe; INS: insulin; tranilast: N-[3,4-dimethoxycinnamonyl]-anthranilic acid; SKF96365: 1-[2-(4-methoxyphenyl)-2-[3-(4-methoxyphenyl)propoxy]ethyl-1H-imidazole.

## Data Availability

This is a narrative review, so a dataset was not created.

## References

[1] Caprodossi S., Lucciarini R., Amantini C. (2008). Transient receptor potential vanilloid type 2 (TRPV2) expression in normal urothelium and in urothelial carcinoma of human bladder: correlation with the pathologic stage. *European Urology*.

[2] Huynh K. W., Cohen M. R., Jiang J. (2016). Structure of the full-length TRPV2 channel by cryo-EM. *Nature Communications*.

[3] Zubcevic L., Herzik M. A., Chung B. C., Liu Z., Lander G. C., Lee S. Y. (2016). Cryo-electron microscopy structure of the TRPV2 ion channel. *Nature Structural & Molecular Biology*.

[4] Caterina M. J., Schumacher M. A., Tominaga M., Rosen T. A., Levine J. D., Julius D. (1997). The capsaicin receptor: a heat-activated ion channel in the pain pathway. *Nature*.

[5] Perálvarez-Marín A., Doñate-Macian P., Gaudet R. (2013). What do we know about the transient receptor potential vanilloid 2 (TRPV2) ion channel?. *The FEBS Journal*.

[6] Huynh K. W., Cohen M. R., Chakrapani S., Holdaway H. A., Stewart P. L., Moiseenkova-Bell V. Y. (2014). Structural Insight into the assembly of TRPV channels. *Structure*.

[7] Santoni G., Farfariello G., Amantini C. (2011). TRPV channels in tumor growth and progression. *Advances in Experimental Medicine and Biology*.

[8] Santoni G., Farfariello V., Liberati S. (2013). The role of transient receptor potential vanilloid type-2 ion channels in innate and adaptive immune responses. *Frontiers in Immunology*.

[9] Nagasawa M., Nakagawa Y., Tanaka S., Kojima I. (2007). Chemotactic peptide fmetleuphe induces translocation of the TRPV2 channel in macrophages. *Journal of Cellular Physiology*.

[10] Link T. M., Park U., Vonakis B. M., Raben D. M., Soloski M. J., Caterina M. J. (2010). TRPV2 has a pivotal role in macrophage particle binding and phagocytosis. *Nature Immunology*.

[11] Issa C. M., Hambly B. D., Wang Y. (2014). TRPV2 in the development of experimental colitis. *Scandinavian Journal of Immunology*.

[12] Yamashiro K., Sasano T., Tojo K. (2010). Role of transient receptor potential vanilloid 2 in LPS-induced cytokine production in macrophages. *Biochemical and Biophysical Research Communications*.

[13] Szöllősi A. G., Oláh A., Tóth I. B. (2013). Transient receptor potential vanilloid-2 mediates the effects of transient heat shock on endocytosis of human monocyte-derived dendritic cells. *FEBS Letters*.

[14] Compan V., Baroja-Mazo A., Lopez-Castejon G. (2012). Cell volume regulation modulates NLRP3 inflammasome activation. *Immunity*.

[15] Sulk M., Seeliger S., Aubert J. (2012). Distribution and expression of non-neuronal transient receptor potential (TRPV) ion channels in rosacea. *The Journal of Investigative Dermatology*.

[16] Nagasawa M., Kojima I. (2012). Translocation of calcium-permeable TRPV2 channel to the podosome: its role in the regulation of podosome assembly. *Cell Calcium*.

[17] Pottosin I., Delgado-Enciso I., Bonales-Alatorre E., Nieto-Pescador M. G., Moreno-Galindo E. G., Dobrovinskaya O. (2015). Mechanosensitive Ca^2+^-permeable channels in human leukemic cells: pharmacological and molecular evidence for TRPV2. *Biochimica et Biophysica Acta*.

[18] Liberati S., Morelli M. B., Amantini C. (2014). Loss of TRPV2 homeostatic control of cell proliferation drives tumor progression. *Cells*.

[19] Morelli M. B., Liberati S., Amantini C. (2013). Expression and function of the transient receptor potential ion channel family in the hematologic malignancies. *Current Molecular Pharmacology*.

[20] Park K. S., Pang B., Park S. J. (2011). Identification and functional characterization of ion channels in CD_34_^+^ hematopoietic stem cells from human peripheral blood. *Molecular Cell*.

[21] Siveen K. S., Prabhu K. S., Parray A. S. (2019). Evaluation of cationic channel TRPV2 as a novel biomarker and therapeutic target in leukemia-implications concerning the resolution of pulmonary inflammation. *Scientific Reports*.

[22] Bai H., Zhu H., Yan Q. (2018). TRPV2 induced Ca^2+^-calcineurin-NFAT signaling regulates differentiation of osteoclast in multiple myeloma. *Cell Communication and Signaling: CCS*.

[23] Morelli M. B., Offidani M., Alesiani F. (2014). The effects of cannabidiol and its synergism with bortezomib in multiple myeloma cell lines. A role for transient receptor potential vanilloid type-2. *International Journal of Cancer*.

[24] Nabissi M., Morelli M. B., Amantini C. (2010). TRPV2 channel negatively controls glioma cell proliferation and resistance to Fas-induced apoptosis in ERK-dependent manner. *Carcinogenesis*.

[25] Alptekin M., Eroglu S., Tutar E. (2015). Gene expressions of TRP channels in glioblastoma multiforme and relation with survival. *Tumor Biology*.

[26] Nabissi M., Morell M. B., Amantini C. (2015). Cannabidiol stimulates Aml-1a-dependent glial differentiation and inhibits glioma stem-like cells proliferation by inducing autophagy in a TRPV2-dependent manner. *International Journal of Cancer*.

[27] Morelli M. B., Nabissi M., Amantini C. (2012). The transient receptor potential vanilloid-2 cation channel impairs glioblastoma stem-like cell proliferation and promotes differentiation. *International Journal of Cancer*.

[28] Gimple R. C., Bhargava S., Dixit D., Rich J. N. (2019). Glioblastoma stem cells: lessons from the tumor hierarchy in a lethal cancer. *Genes & Development*.

[29] Santoni G., Amantini C. (2019). The transient receptor potential vanilloid type-2(TRPV2) ion channels in neurogenesis and gliomagenesis: cross-talk between transcription factors and signaling molecules. *Cancers*.

[30] Katanosaka K., Takatsu S., Mizumura K., Naruse K., Katanosaka Y. (2018). TRPV2 is required for mechanical nociception and the stretch-evoked response of primary sensory neurons. *Scientific Reports*.

[31] Aguado T., Carracedo A., Julien B. (2007). Cannabinoids induce glioma stem-like cell differentiation and inhibit gliomagenesis. *The Journal of Biological Chemistry*.

[32] McAllister S. D., Murase R., Christian R. T. (2011). Pathways mediating the effects of cannabidiol on the reduction of breast cancer cell proliferation, invasion, and metastasis. *Breast Cancer Research and Treatment*.

[33] Nabissi M., Morelli M. B., Santoni M., Santoni G. (2013). Triggering of the TRPV2 channel by cannabidiol sensitizes glioblastoma cells to cytotoxic chemotherapeutic agents. *Carcinogenesis*.

[34] Gong X., Schwartz P. H., Linskey M. E., Bota D. A. (2011). Neural stem/progenitors and glioma stem-like cells have differential sensitivity to chemotherapy. *Neurology*.

[35] Torres S., Lorente M., Rodriguez-Fornes F. (2011). A combined preclinical therapy of cannabinoids and temozolomide against glioma. *Molecular Cancer Therapeutics*.

[36] Dall'Stella P. B., Docema M. F. L., Maldaun M. V. C., Feher O., Lancellotti C. L. P. (2019). Case report: clinical outcome and image response of two patients with secondary high-grade glioma treated with chemoradiation, PCV, and cannabidiol. *Frontiers in Oncology*.

[37] Santoni G., Amantini C., Maggi F. (2020). The TRPV2 cation channels: from urothelial cancer invasiveness to glioblastoma multiforme interactome signature. *Laboratory Investigation*.

[38] Donate-Macian P., Gomez A., Degano I. R., Peralvarez-Marin A. (2018). A TRPV2 interactome-based signature for prognosis in glioblastoma patients. *Oncotarget*.

[39] Subramaniam V., Ace O., Prud'homme G. J., Jothy S. (2011). Tranilast treatment decreases cell growth, migration and inhibits colony formation of human breast cancer cells. *Experimental and Molecular Pathology*.

[40] Nie L., Oishi Y., Doi I., Shibata H., Kojima I. (1997). Inhibition of proliferation of MCF-7 breast cancer cells by a blocker of Ca^2+^-permeable channel. *Cell Calcium*.

[41] Chakrabarti R., Subramaniam V., Abdalla S., Jothy S., Prudʼhomme G. J. (2009). Tranilast inhibits the growth and metastasis of mammary carcinoma. *Anti-Cancer Drugs*.

[42] Gambade A., Zreika S., Guéguinou M. (2016). Activation of TRPV2 and BKCa channels by the LL-37 enantiomers stimulates calcium entry and migration of cancer cells. *Oncotarget*.

[43] Méndez-Samperio P. (2010). The human cathelicidin hCAP18/LL-37: a multifunctional peptide involved in mycobacterial infections. *Peptides*.

[44] Elbaz M., Ahirwar D., Xiaoli Z. (2018). TRPV2 is a novel biomarker and therapeutic target in triple negative breast cancer. *Oncotarget*.

[45] Zhou K., Zhang S.-S., Yan Y., Zhao S. (2014). Overexpression of transient receptor potential vanilloid 2 is associated with poor prognosis in patients with esophageal squamous cell carcinoma. *Medical Oncology*.

[46] Kudou M., Shiozaki A., Yamazato Y. (2019). The expression and role of TRPV2 in esophageal squamous cell carcinoma. *Scientific Reports*.

[47] Shiozaki A., Kudou M., Ichikawa D. (2018). Esophageal cancer stem cells are suppressed by tranilast, a TRPV2 channel inhibitor. *Journal of Gastroenterology*.

[48] Bray F., Ferlay J., Soerjomataram I., Siegel R. L., Torre L. A., Jemal A. (2018). Global cancer statistics 2018: GLOBOCAN estimates of incidence and mortality worldwide for 36 cancers in 185 countries. *CA: a Cancer Journal for Clinicians*.

[49] Van Cutsem E., Sagaert X., Topal B., Haustermans K., Prenen H. (2016). Gastric cancer. *Lancet*.

[50] Zoppoli P., Calice G., Laurino S. (2019). TRPV2 calcium channel gene expression and outcomes in gastric cancer patients: a clinically relevant association. *Journal of Clinical Medicine*.

[51] Liu G., Xie C., Sun F. (2010). Clinical significance of transient receptor potential vanilloid 2 expression in human hepatocellular carcinoma. *Cancer Genetics and Cytogenetics*.

[52] Ma W., Li C., Yin S. (2015). Novel role of TRPV2 in promoting the cytotoxicity of H_2_O_2_-mediated oxidative stress in human hepatoma cells. *Free Radical Biology & Medicine*.

[53] Liu L.-L., Fu D., Ma Y., Shen X.-Z. (2011). The power and the promise of liver cancer stem cell markers. *Stem Cells and Development*.

[54] Huang R., Wang F., Yang Y. (2019). Recurrent activations of transient receptor potential vanilloid-1 and vanilloid-4 promote cellular proliferation and migration in esophageal squamous cell carcinoma cells. *FEBS Open Bio*.

[55] Hu Z., Cao X., Fang Y. (2018). Transient receptor potential vanilloid-type 2 targeting on stemness in liver cancer. *Biomedicine & Pharmacotherapy*.

[56] Yamada T., Ueda T., Shibata Y. (2010). TRPV2 activation induces apoptotic cell death in human T24 bladder cancer cells: a potential therapeutic target for bladder cancer. *Urology*.

[57] Mizuno H., Suzuki Y., Watanabe M. (2014). Potential role of transient receptor potential (TRP) channels in bladder cancer cells. *The Journal of Physiological Sciences*.

[58] Liu Q., Wang X. (2013). Effect of TRPV2 cation channels on the proliferation, migration and invasion of 5637 bladder cancer cells. *Experimental and Therapeutic Medicine*.

[59] Oulidi A., Bokhobza A., Gkika D. (2013). TRPV2 mediates adrenomedullin stimulation of prostate and urothelial cancer cell adhesion, migration and invasion. *PLoS One*.

[60] Wang C., Hu H. Z., Colton C. K., Wood J. D., Zhu M. X. (2004). An alternative splicing product of the murine *trpv*1 gene dominant negatively modulates the activity of TRPV1 channels. *The Journal of Biological Chemistry*.

[61] Zhao H., Grossman H. B., Spitz M. R., Lerner S. P., Zhang K., Wu X. (2003). Plasma levels of insulin like growth factor-1 and binding protein-3, and their association with bladder cancer risk. *The Journal of Urology*.

[62] Monet M., Gkika D., Lehen'kyi V. (2009). Lysophospholipids stimulate prostate cancer cell migration via TRPV2 channel activation. *Biochimica et Biophysica Acta*.

[63] Monet M., Lehen'kyi V., Gackiere F. (2010). Role of cationic channel TRPV2 in promoting prostate cancer migration and progression to androgen resistance. *Cancer Research*.

[64] Sugio S., Nagasawa M., Kojima I., Ishizaki Y., Shibasaki K. (2017). Transient receptor potential vanilloid 2 activation by focal mechanical stimulation requires interaction with the actin cytoskeleton and enhances growth cone motility. *The FASEB Journal*.

[65] Warrington J. I., Richards G. O., Wang N. (2017). The role of the calcitonin peptide family in prostate cancer and bone metastasis. *Current Molecular Biology Reports*.

[66] Fusi C., Materazzi S., Minocci D. (2014). Transient receptor potential vanilloid 4 (TRPV4) is downregulated in keratinocytes in human non-melanoma skin cancer. *The Journal of Investigative Dermatology*.

[67] Zheng J., Liu F., Du S. (2019). Mechanism for Regulation of Melanoma Cell Death via Activation of Thermo-TRPV4 and TRPV2. *Journal of Oncology*.

